# Not All Gastric Air Is Equal: A Case Report Highlighting the Difficulty in Distinguishing Between Benign and Lethal Gastric Pneumatosis

**DOI:** 10.7759/cureus.113059

**Published:** 2026-07-20

**Authors:** Asanka B Ekanayake, Emil S Oweis

**Affiliations:** 1 Internal Medicine, Walter Reed National Military Medical Center, Bethesda, USA; 2 Pulmonary and Critical Care Medicine, MedStar Washington Hospital Center, Washington, USA

**Keywords:** emphysematous gastritis, gastric emphysema, gastric pneumatosis, out of hospital cardiac arrest, percutaneous endoscopic gastrostomy dependence

## Abstract

Gastric pneumatosis is a rare radiologic finding characterized by intramural gas within the gastric wall and includes two entities, gastric emphysema (GE) and emphysematous gastritis (EG). GE is typically benign and noninfectious, whereas EG results from invasion by gas-forming organisms and carries high mortality. Distinguishing between them can be challenging, particularly in critically ill patients. A 34-year-old man with autism spectrum disorder, severe protein-calorie malnutrition, and percutaneous endoscopic gastrostomy (PEG) dependence presented in shock following out-of-hospital cardiopulmonary arrest requiring prolonged resuscitation. CT imaging demonstrated right lower lobe pneumonia and gastric pneumatosis remote from the PEG site. He had no abdominal symptoms. Esophagogastroduodenoscopy revealed intact gastric mucosa without ischemia or necrosis. Blood cultures grew pan-susceptible *Escherichia coli*. With supportive care and antibiotics, he rapidly stabilized, and repeat imaging within 48 hours showed complete resolution. Despite bacteremia and shock, preserved mucosal integrity and rapid radiographic improvement supported gastric emphysema, illustrating the importance of integrating clinical, radiographic, and endoscopic findings to guide management and avoid unnecessary surgery.

## Introduction

Gastric emphysema is a rare radiologic finding characterized by the presence of intramural gas within the stomach wall and represents an uncommon imaging abnormality with varying clinical significance. Gastric pneumatosis can be broadly categorized into gastric emphysema (GE) and emphysematous gastritis (EG). GE refers to noninfectious intramural air within the gastric wall without underlying infection, whereas EG results from transmural invasion by gas-forming organisms and is typically associated with systemic toxicity and high mortality [1-3].

The pathogenesis of GE is not fully understood, but it is thought to be caused by a breach in the gastric wall's integrity. This breach can be caused by a variety of factors, including ischemic injury during systemic hypoperfusion, barotrauma from cardiopulmonary resuscitation, vomiting or positive-pressure ventilation, iatrogenic mucosal disruption, and elevated intragastric pressure from obstruction or feeding devices [4-6]. In contrast, EG arises from bacterial invasion of compromised gastric mucosa, most commonly involving *Escherichia coli*, *Streptococcus species*, *Enterobacter*, or *Pseudomonas aeruginosa* [3,7]. Although the stomach's acidic environment and robust vascular supply ordinarily protect against infection, these defenses may be impaired in the setting of critical illness, malnutrition, or mucosal injury [2,8]. Clinically, GE is often incidental and self-limited, whereas EG presents with severe abdominal pain, fever, leukocytosis, metabolic derangements, and hemodynamic instability [3,9-10].

Radiographic findings alone are insufficient to distinguish these entities, necessitating integration of clinical context, laboratory data, and endoscopic evaluation [1]. Early differentiation is essential, as inappropriate classification may expose patients to unnecessary surgery or delay potentially life-saving intervention. This case underscores the diagnostic complexity of this entity, demonstrating that differentiation between GE and EG can remain difficult despite early evaluation, particularly in the presence of overlapping clinical features and concurrent risk factors including mechanical barotrauma and systemic infection.

## Case presentation

A 34-year-old male with a history of autism spectrum disorder, hypothyroidism, asthma, recurrent pneumonia, and severe protein-calorie malnutrition requiring enteral nutrition through a chronic percutaneous endoscopic gastrostomy (PEG) tube placed over one year prior presented to the hospital by emergency medical services (EMS) after his mother found him unresponsive with faint pulses in his bedroom. His mother started cardiopulmonary resuscitation (CPR) and called EMS. CPR was performed for a maximum of 15 minutes before EMS arrived. While en route to the hospital, the patient was hypotensive; however, pulses were still palpable, and IV fluids were started.

**Figure 1:**
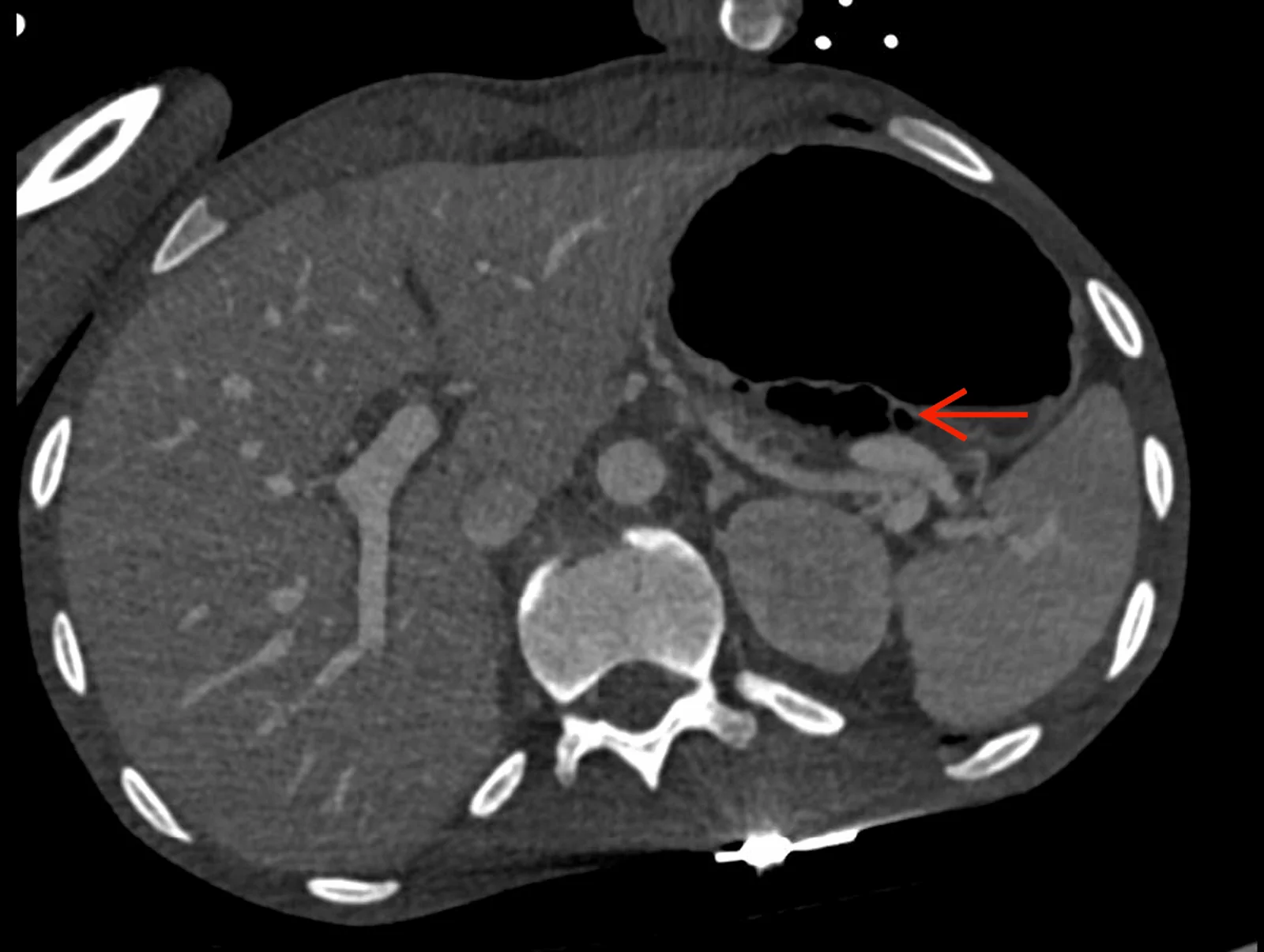
Non-contrast-enhanced CT scan of the abdomen and pelvis demonstrating intramural gas along the gastric wall, consistent with gastric pneumatosis Arrow depicts intramural gas along the gastric wall.

**Figure 2:**
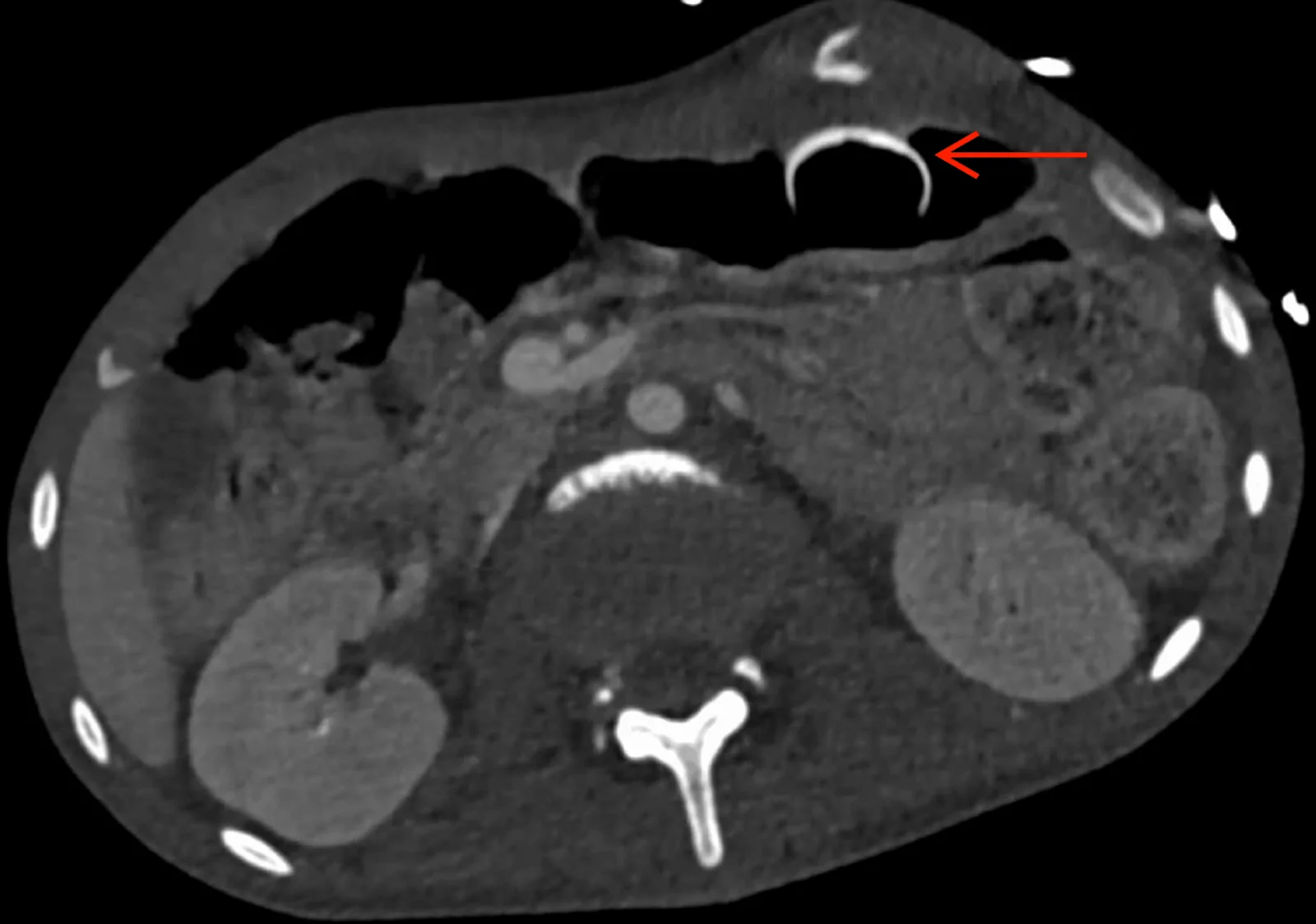
Non-contrast-enhanced CT of the abdomen and pelvis demonstrating a previously placed PEG tube within the stomach Arrow depicts percutaneous endoscopic gastrostomy (PEG) tube within the stomach.

On arrival to the hospital, the patient remained hypotensive and encephalopathic, unable to provide a history. His mother provided initial history and stated that the day prior, the patient was in his normal health and was communicative at baseline. She denied him complaining of any nausea or vomiting, abdominal pain, melena, or hematochezia. Initial vitals: patient was febrile to 39.7 degrees Celsius, tachycardic to 178 bpm, and blood pressure (BP) was 45/28 mmHg. Initial laboratory examination was notable for lactic acid at 2.2 mmol/L, sodium of 166 mmol/L, creatinine at 4 mg/dL, from a baseline of 0.9 mg/dL, elevated anion gap, high-sensitivity troponins elevated to 230 ng/mL, thyroid-stimulating hormone at 9.5 mIU/L, and aspartate aminotransferase (AST) to 60 U/L. Continuous intravenous fluids were started, and peripheral norepinephrine was started at the maximum dose. Due to acute altered mental status and inability to protect airway, the patient was intubated. The patient's blood pressure began increasing, and norepinephrine was titrated to maintain mean arterial pressure greater than 90/60 mmHg. Blood and urine cultures were collected, and he was started on broad-spectrum antibiotics. Initial imaging, including point-of-care ultrasound, was negative for any trauma or intra-abdominal bleeding. Computed tomography (CT) of the chest, abdomen, and pelvis was notable for consolidation in the right lower lobe of the lung and gastric pneumatosis on the opposite side of his previously placed percutaneous endoscopic gastrostomy (PEG) tube (Figures 1, 2). Physical exam of his abdomen was scaphoid, soft, non-distended, and no erythema was noted at the PEG site. General surgery and gastroenterology were consulted for endoscopic evaluation of gastric pneumatosis. 

An esophagogastroduodenoscopy (EGD) was performed approximately two hours after the initial CT scan and showed the gastric mucosa appeared smooth, pink to reddish-appearing, and well perfused with preserved rugal architecture and an intact subepithelial vascular pattern. No pallor, dusky discoloration, necrosis, friability, erosive changes, ulcerative inflammation, or evidence of ischemic injury was identified. The cardia and gastroesophageal junction similarly demonstrated healthy-appearing mucosa. A small gastric ulcer was noted in the body of the stomach. 

**Figure 3:**
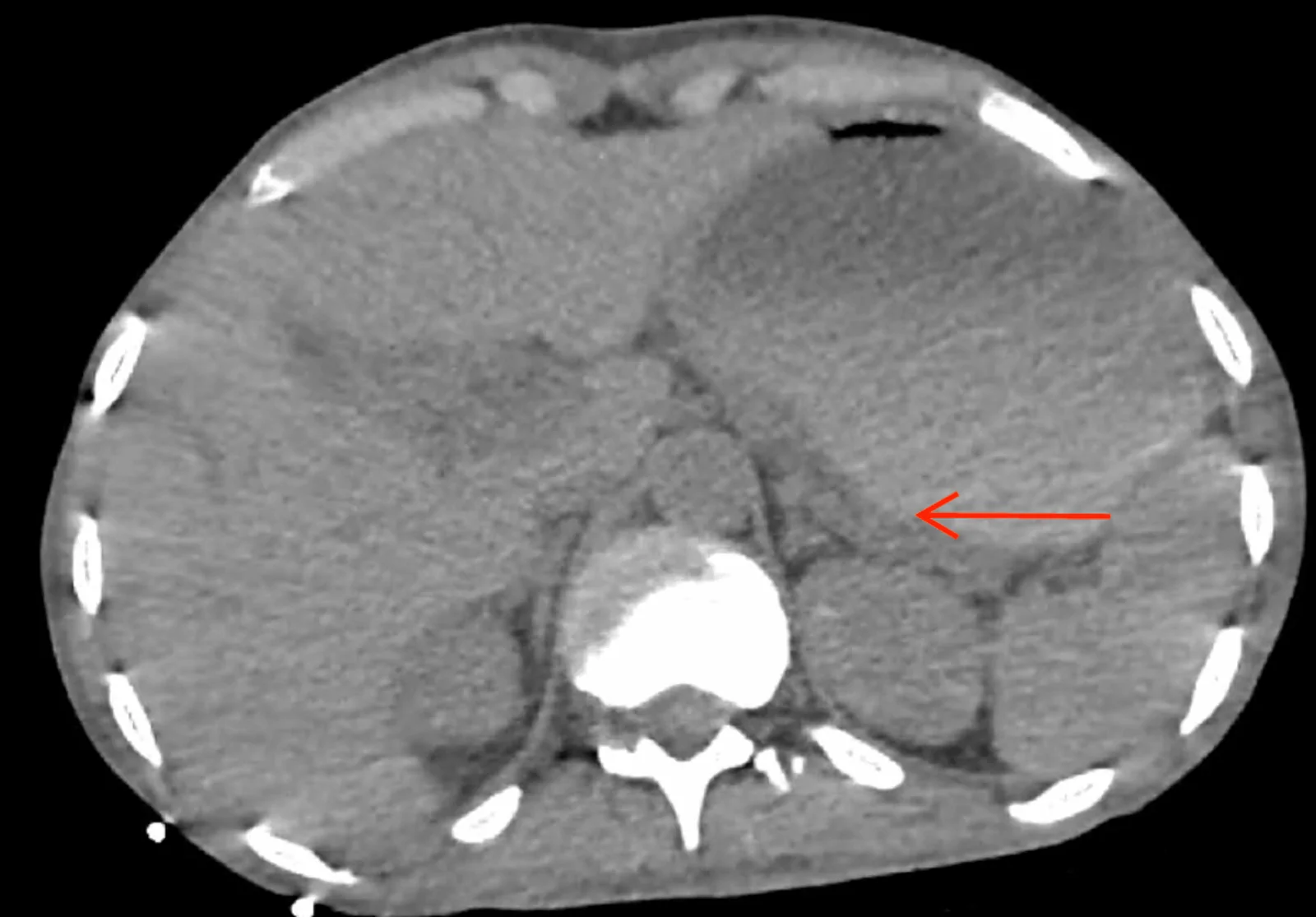
Non-contrast CT of the abdomen obtained 48 hours after admission demonstrating resolution of previously identified gastric pneumatosis Arrow depicts the resolution of gastric free air.

**Figure 4:**
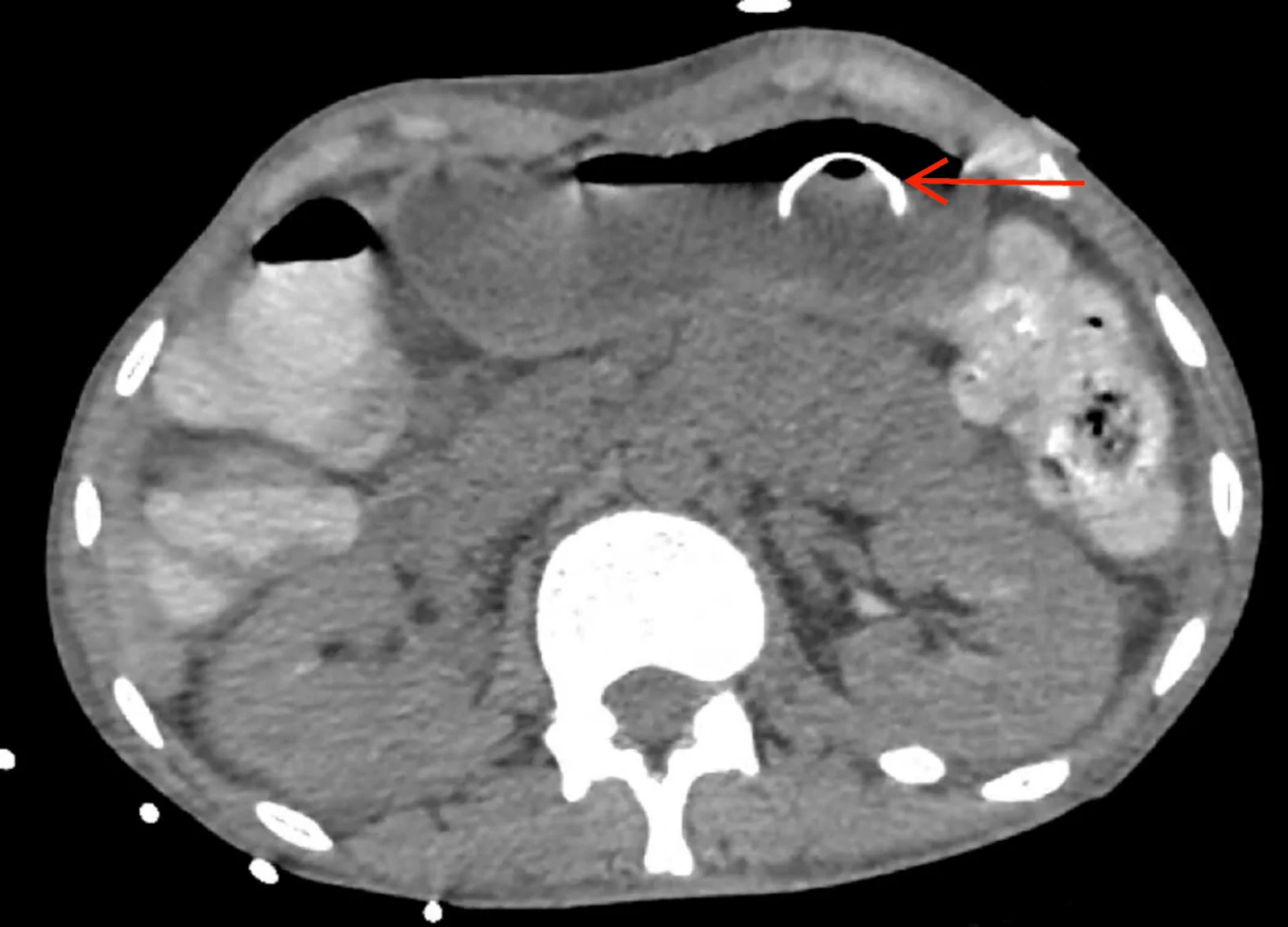
Non-contrast CT of the abdomen obtained 48 hours after admission demonstrating an PEG tube in appropriate position Arrow depicts a percutaneous endoscopic gastrostomy (PEG) tube within the stomach.

After discussion with gastroenterology and general surgery teams, the ulcer appeared to be an incidental finding and likely not the contributing factor to this presentation. The patient was transferred to the intensive care unit. Over the subsequent 24 hours, the patient's vasopressor requirement was weaned completely down, and the patient passed a spontaneous breathing trial and was subsequently extubated. The patient's PEG tube was not removed, and feeds were restarted. Blood cultures resulted in pan-susceptible *Escherichia coli*, and broad-spectrum antibiotics were switched to ceftriaxone, which was continued for seven days. A repeat CT scan of the abdomen 48 hours after admission showed resolution of gastric pneumatosis (Figures 3, 4). The patient was transferred to the medicine floor due to clinical stability. Table 1 summarizes the patient's clinical course.

**Table 1:** Timeline summarizing the patient's presentation, diagnostic evaluation, treatment, and rapid clinical and radiographic resolution CPR - cardiopulmonary resuscitation; EMS - emergency medical services

Time	Clinical Course	Diagnostic Workup	Interventions
Day prior to presentation	At baseline health per mother; communicative; no nausea, vomiting, abdominal pain, melena, or hematochezia.	—	—
Out-of-hospital event	Found unresponsive with faint pulses. CPR initiated by mother and continued for up to 15 minutes before EMS arrival.	—	CPR performed; IV fluids initiated by EMS for hypotension.
Emergency department (0 hours)	Febrile (39.7°C), tachycardic (178 bpm), profound hypotension (45/28 mmHg), encephalopathic, acute respiratory failure. Abdomen soft, scaphoid, non-distended, without PEG-site erythema or peritoneal signs.	Laboratory studies notable for lactate 2.2 mmol/L, sodium 166 mmol/L, creatinine 4.0 mg/dL (baseline 0.9 mg/dL), elevated anion gap, hs-troponin 230 ng/mL, TSH 9.5 mIU/L, AST 60 U/L. Blood and urine cultures obtained.	Intravenous fluids, broad-spectrum antibiotics, norepinephrine, endotracheal intubation.
Initial imaging (0 hours)	Gastric pneumatosis identified incidentally during evaluation for shock.	CT chest/abdomen/pelvis demonstrated right lower lobe consolidation and gastric pneumatosis remote from the PEG insertion site.	Gastroenterology and general surgery consulted.
~2 hours after CT	No abdominal symptoms or evidence of peritonitis.	EGD demonstrated preserved gastric mucosa with intact rugal folds and vascular pattern, without ischemia or necrosis. Small incidental gastric body ulcer identified.	Continued with broad-spectrum antibiotics, norepinephrine, endotracheal intubation.
First 24 hours	Rapid hemodynamic improvement.	Blood cultures positive for pan-susceptible Escherichia coli.	Vasopressors discontinued, extubated after successful spontaneous breathing trial, antibiotics narrowed to Ceftriaxone.
48 hours after admission	Clinically stable.	Repeat CT abdomen demonstrated complete resolution of gastric pneumatosis.	Transferred from ICU to medical floor for continued care.

## Discussion

Overall, when presented with a patient who has intramural air on initial imaging, it is important to consider both GE and EG as possible differential diagnoses. The diagnosis and management algorithms for these conditions differ significantly. 

In patients with gastric pneumatosis, we suggest a structured evaluation that incorporates clinical, laboratory, radiographic, and endoscopic findings. When gastric pneumatosis is identified or suspected, evaluation should begin with assessment of the patient's clinical presentation, hemodynamic status, abdominal examination findings, and laboratory studies, including a complete blood count, comprehensive metabolic panel, and serum lactate. These findings help determine the likelihood of a benign versus life-threatening process and guide the urgency of further investigation. Cross-sectional imaging with contrast-enhanced CT of the abdomen and pelvis should then be obtained to characterize the extent and distribution of intramural gas and identify associated findings such as portal venous gas, perforation, or ischemia. 

Endoscopic evaluation should be considered when the diagnosis remains uncertain after clinical and radiographic assessment or when there is concern for ischemia, necrosis, or other mucosal pathology. While gastric emphysema and emphysematous gastritis are both intramural processes, endoscopy may provide important information regarding mucosal viability. Findings that favor emphysematous gastritis include mucosal pallor, friability, ulceration, erosions, necrosis, or diffuse inflammatory changes. On the other hand, preservation of normal vascular patterns, intact rugal folds, and absence of ischemic or necrotic changes support a more benign process such as gastric emphysema. Endoscopic findings should be interpreted in conjunction with the clinical presentation and imaging findings, as no single modality is independently diagnostic.

From a treatment perspective, EG involves correcting any acid-base or electrolyte imbalances, administering intravenous antimicrobial therapy covering gram-negative organisms and anaerobic organisms, bowel rest, and providing fluid resuscitation [2,11]. When expectant medical management fails, definitive surgical treatment with gastrectomy may be necessary. But this should only be done when there is evidence of ischemic bowel or necrosis, as surgery without these indications poses a high risk of bowel perforation [3,9]. Thus, it is possible to delay the definitive treatment for the patient until they are clinically stable and their mucosa is less friable [3,9]. However, despite receiving early intervention, the mortality rate for EG remains above 50% [1,10]. 

Moreover, research has indicated that serum lactate levels greater than 2.0 mmol/L at the time of diagnosis are associated with increased mortality in patients with gastric pneumatosis [12]. Notably, our patient's lactate was 2.2 mmol/L, exceeding this threshold despite complete clinical recovery and rapid radiographic resolution without surgical intervention. In this case, lactate elevation likely reflected systemic hypoperfusion in the setting of profound septic shock rather than transmural gastric ischemia. This observation highlights an important limitation of using serum lactate in isolation and reinforces the need to interpret laboratory abnormalities within the broader clinical, radiographic, and endoscopic context.

If EG is ruled out, conservative management of GE is typically sufficient [1,13]. The primary management steps should focus on supportive medical care and treating the underlying condition (such as gastric outlet obstruction or mucosal disruption), and close observation [10,14]. Antimicrobial therapy is generally not required unless there is concern for secondary infection [10,15]. Surgical intervention is rarely indicated in GE; the primary indication for surgery is diagnostic uncertainty when EG cannot be definitively excluded [2].

This case illustrates the diagnostic challenge posed by gastric pneumatosis in a critically ill, young adult. Our patient presented in profound septic shock of unknown origin with multiorgan dysfunction. Gastric pneumatosis was identified incidentally on computed tomography in the absence of abdominal symptoms or peritoneal signs. Endoscopic evaluation demonstrated healthy gastric mucosa with preserved rugal folds and intact capillary networks, largely excluding EG and supporting a diagnosis of GE. 

However, a limitation of this report is the absence of endoscopic photographic documentation. The endoscopy was performed as an urgent assessment of mucosal viability in a critically ill patient, and no images were obtained during the procedure. Consequently, interpretation relies on contemporaneous procedural documentation and multidisciplinary review rather than direct visual confirmation.

Several mechanisms may have contributed to the development of gastric emphysema in this patient, including systemic hypoperfusion during shock, barotrauma from cardiopulmonary resuscitation, subsequent mechanical ventilation, and gastric insufflation during bag-valve-mask ventilation [16-20]. Other contributing factors are possible, although the supporting evidence is less direct. GE has been reported in patients with anorexia nervosa and severe malnutrition [21], with proposed mechanisms including mucosal atrophy and increased mucosal permeability [18]. While protein-calorie malnutrition has not been identified as a direct cause of gastric emphysema, it may have increased this patient's susceptibility to intramural gas formation through impaired mucosal integrity.

Similarly, PEG tubes have been associated with instrumentation-related mucosal injury and pneumoperitoneum [22]; however, PEG tubes have not been well described as a direct cause of gastric emphysema [18]. In our patient, the intramural gas was located remote from the PEG insertion site, and no PEG-site abnormalities or surrounding inflammatory changes were identified on imaging or physical examination, making a direct PEG-related etiology less likely.

Finally, inadvertent esophageal intubation is a recognized cause of gastric emphysema due to excessive gastric insufflation and elevated intragastric pressures [20]. Although no such event was documented during this patient's resuscitation, it cannot be completely excluded as a contributing factor.

Despite the reassuring endoscopic appearance and rapid radiographic resolution, the precise etiology of this patient's gastric pneumatosis remains uncertain. This case uniquely walks the line of the traditional dichotomy between benign gastric emphysema and emphysematous gastritis. On one hand, the patient experienced significant barotrauma in the setting of initial cardiopulmonary resuscitation and subsequent mechanical ventilation, which are well-recognized mechanisms for noninfectious intramural air [8,16-18]. On the other hand, his presentation was complicated by *Escherichia coli* bacteremia, an organism implicated in emphysematous gastritis [23]. 

These opposing features underscore the diagnostic ambiguity that can arise even when endoscopy demonstrates preserved mucosal integrity. While the absence of mucosal ischemia, necrosis, or friability strongly argued against emphysematous gastritis, the coexistence of systemic infection precludes definitive attribution to a purely benign process. This overlap highlights that gastric pneumatosis may not always conform neatly to established categories and reinforces the importance of integrating radiographic findings with clinical trajectory and endoscopic assessment rather than relying on any single modality.

## Conclusions

Gastric pneumatosis is a rare but clinically significant radiographic finding that spans a spectrum from benign gastric emphysema to life-threatening emphysematous gastritis. This case illustrates the diagnostic ambiguity that arises when features of both entities coexist. These opposing elements precluded definitive attribution to a single pathogenic mechanism and highlight the limitations of relying on any single diagnostic modality. Accurate interpretation of gastric pneumatosis requires integration of imaging, laboratory data, endoscopic assessment, and most importantly, the patient's clinical trajectory. Early multidisciplinary evaluation and individualized management can prevent unnecessary surgical intervention while ensuring timely escalation of care when warranted, reinforcing that gastric pneumatosis should be approached as a dynamic clinical entity rather than a fixed diagnosis.
